# Robotic exoskeleton rehabilitation in incomplete L2 paraplegia: functional gains contrast with metabolic challenges – a multidimensional case report

**DOI:** 10.3389/fresc.2026.1616983

**Published:** 2026-04-10

**Authors:** Rafael Francisco Vieira de Melo, Daniela Mitiyo Odagiri Utiyama, Priscila Fabiano Carvalho, André Tadeu Sugawara, Linamara Rizzo Battistella

**Affiliations:** 1 Instituto de Medicina Fisica e Reabilitacao, Hospital das Clinicas HCFMUSP, Faculdade de Medicina, Universidade de Sao Paulo, Sao Paulo, SP, Brazil; 2 Departamento de Medicina Legal, Bioetica, Medicina do Trabalho e Medicina Fisica e Reabilitacao, Faculdade de Medicina FMUSP, Universidade de Sao Paulo, Sao Paulo, SP, Brazil

**Keywords:** bone densitometry, case report, isokinetic dynamometry, rehabilitation, robotic exoskeleton, spinal cord injury

## Abstract

**Introduction:**

Incomplete paraplegia presents complex rehabilitation challenges due to its multisystemic impact on motor function, bone health, and metabolic regulation. This case report evaluates the effects of robotic exoskeleton rehabilitation addressing a critical gap in understanding the multidimensional outcomes of this emerging technology.

**Case presentation:**

An 18-year-old male with traumatic L2 AIS C paraplegia underwent 20 sessions of robotic exoskeleton training (Atalante exoskeleton) during 60 min of therapy (20-min setup, 20-min walking, 10-min squats and recovery) combined with conventional physiotherapy over six months. Conventional therapy complemented exoskeleton training by addressing residual strength deficits through resisted knee extension (3 × 10 reps at 60% 1-RM) and functional mobility via overground gait training with knee-ankle-foot orthoses (KAFOs) (2×/week, 10 m walks. Comprehensive assessments included isokinetic dynamometry (CYBEX), bone densitometry (DXA), AIS impairment scale, and functional mobility tests (Timed Up and Go, 10-meter walk test). Results demonstrated neurological improvement (AIS L2–L3 motor score improved from 4/5 to 5/5, with light touch improving from 78 to 84) and gait capacity recovery (300 m with crutches). Musculoskeletal outcomes revealed a 21.7% improvement in right hip extensor torque (69→84 Nm), though still 19.2% below the patient's own 6-month post-injury baseline value (104 Nm), while bone mineral density showed site-specific effects (lumbar spine: +50%, T-score −2.6→0.8; femur: T-score −3.3→−1.3). Notably, functional gains correlated with hip torque recovery (TUG: r = −0.71), but bilateral deficit worsened (12%→42%), and visceral fat increased dramatically (359%) despite functional improvements.

**Discussion:**

This case demonstrates that exoskeleton training effectively enhances mobility and bone health through weight-bearing stimulation, particularly at lumbar spine. However, the paradoxical metabolic changes and persistent neuromuscular asymmetries reveal critical limitations of current robotic rehabilitation approaches. These findings support the concept of spinal cord injury as a multisystem disorder requiring integrated interventions that combine exoskeleton training with nutritional strategies and asymmetric muscle training. The case provides novel insights for developing comprehensive rehabilitation protocols that address both functional recovery and secondary health complications in incomplete paraplegia.

## 1 Introduction

Spinal cord injury (SCI) represents one of the most complex neurological conditions in modern medicine, with consequences that extend far beyond motor impairment to affect multiple physiological systems. The American Spinal Injury Association Impairment Scale (AIS) (1), recently updated in 2023, provides clinicians with a refined tool for assessing neurological deficits, particularly for incomplete injuries (AIS C and D) where subtle preservation of sensory and autonomic function can significantly influence rehabilitation outcomes. AIS classification is based primarily on motor function; in this specific case, sensory preservation was documented separately as absent below L2, though later assessment indicated intact sensation up to L3 with hypesthesia below. Our understanding of SCI has evolved dramatically in recent years, with growing recognition that it represents not just a localized injury but rather a systemic disorder involving persistent neuroinflammation, metabolic dysregulation, and autonomic dysfunction that contribute to various secondary complications. This paradigm shift has fundamentally changed our approach to rehabilitation, moving from a narrow focus on motor recovery to a more comprehensive strategy that addresses the multiple physiological systems affected by the injury (2).

The development of robotic exoskeletons has marked a significant advancement in neurorehabilitation, offering new hope for patients with SCI. These motorized wearable devices have demonstrated potential not only for restoring mobility but also for inducing neuroplastic changes (3). Recent research using advanced neuroimaging techniques has revealed that exoskeleton-assisted walking can activate sensorimotor networks even in chronic SCI (4), suggesting these devices may facilitate neural reorganization.

Robotic exoskeletons show promise for gait rehabilitation in SCI, current literature predominantly focuses on motor outcomes in heterogeneous populations. This case provides unique multidimensional data on a single patient with traumatic L2 AIS C paraplegia, systematically tracking: (1) site-specific bone density changes, (2) simultaneous functional gains and metabolic deterioration, and (3) evolving neuromuscular asymmetry - a triad rarely documented together in existing exoskeleton studies. Our longitudinal design (6-month intervention with 4 assessment timepoints) offers granular insights into the temporal progression of these interrelated outcomes (3).

Perhaps even more intriguing are recent metabolomic studies showing that exoskeleton training leads to measurable changes in encouraging results, including alterations in myokine profiles and bone turnover markers. These findings indicate that the benefits of exoskeleton therapy may extend well beyond the obvious improvements in mobility and muscle strength, potentially influencing multiple physiological systems simultaneously (5).

Assessment of body composition has emerged as a critical component in the comprehensive management of SCI. Current clinical guidelines strongly recommend regular monitoring using dual-energy x-ray absorptiometry (DXA) due to the rapid and significant bone mineral density loss that typically occurs following injury (5–7). This bone loss, combined with the characteristic changes in body composition seen in SCI patients, creates a complex clinical picture that requires careful monitoring and intervention. The integration of isokinetic dynamometry (CYBEX) (8) into rehabilitation protocols provides clinicians with objective, quantifiable measures of muscle performance, which are particularly valuable for predicting functional recovery potential. These advanced assessment tools, when used in combination, enable a more comprehensive evaluation of rehabilitation outcomes that goes beyond traditional functional measures and provides deeper insight into the physiological changes occurring during recovery (2, 3, 5).

These clinical observations align with developing neurophysiological models that propose rhythmic mechanical input from weight-bearing activities may influence autonomic function through stimulation of dorsal root ganglion signaling pathways – a hypothesis extrapolated from broader literature (5). This suggests that exoskeleton therapy may exert its benefits through multiple mechanisms, some of which we are only beginning to understand.

This detailed case report applies the most current CARE guidelines to present a comprehensive evaluation of exoskeleton rehabilitation in a patient with incomplete L2 paraplegia (AIS C). Our assessment incorporates the latest DXA protocols for body composition analysis, advanced CYBEX-derived muscle performance metrics, continuous physiological monitoring data, and novel autonomic function assessments. The findings contribute significantly to our evolving understanding of SCI as a multisystem disorder that requires integrated, multidisciplinary rehabilitation approaches. Perhaps most importantly, this case highlights how emerging rehabilitation technologies like exoskeletons may simultaneously target multiple affected physiological systems through mechanisms that bridge the gap between basic neuroscience and clinical application. As research in this field continues to advance, we are gaining unprecedented insights into the complex interplay between mechanical stimulation, neural plasticity, and systemic physiological regulation in the context of SCI rehabilitation.

The objective of the study is to evaluate the integrated effects of robotic exoskeleton rehabilitation on motor function, body composition, and musculoskeletal parameters in a patient with incomplete L2 paraplegia (AIS C), using objective assessments through isokinetic dynamometry (CYBEX) and bone densitometry (DXA).

## 2 Methodology

### 2.1 Study design

This study consists of a prospective longitudinal case report, developed in accordance with the CARE (CAse REport) guidelines, which provide a standardized framework for clinical case reports with high scientific value. The longitudinal design enabled sequential and integrated assessment of multiple physiological and functional parameters over a 6-month intervention period, with four well-defined evaluation time points: T0 (baseline, 6 months post-injury), T1 (pre-intervention, ∼9 months post-injury), T2 (mid-intervention, ∼12 months post-injury), and T3 (post-intervention, ∼13 months post-injury). This follows current methodological recommendations for spinal cord injury rehabilitation studies (5). The prospective approach ensured rigorous control of data collection, minimizing recall bias and guaranteeing standardized assessment protocols.

The study was conducted at Instituto de Medicina física e reabilitação do Hospital das Clínicas da Faculdade de Medicina da Universidade de São Paulo (HCFMUSP), a national reference center for neurological rehabilitation. The research protocol was approved by the institution's Research Ethics Committee (CAAE: 80753424.6.0000.0068; Approval Number: 6,952,225), in compliance with Brazilian National Health Council Resolution 466/2012 and the ethical principles of the Declaration of Helsinki. All procedures were thoroughly explained to the patient and his legal guardian, who provided written informed consent, including specific authorization for the use of images and clinical data for scientific publication purposes.

### 2.2 Participant

The study case involves an 18-year-old male patient diagnosed with incomplete paraplegia at the L2 level (second lumbar segment of the spinal cord), classified as AIS C (American Spinal Injury Association Impairment Scale Grade C) secondary to a gunshot wound trauma occurring in January 2023. The injury was confirmed by spinal Magnetic resonance imaging (MRI), which revealed a spinal cord contusion at the L1 level with partial preservation of sensory and motor pathways below the neurological level. Baseline assessment (T0) occurred at 6 months post-injury (July 2023). Subsequent assessments were conducted as follows: T1 (pre-intervention) at approximately 9 months post-injury, T2 (mid-intervention) at 12 months post-injury, and T3 (post-intervention) at 13 months post-injury. The period between T0 (Baseline) and T1 (Pre-Intervention) consisted of routine conventional physiotherapy without robotic exoskeleton training. Percentage change (Δ%) is calculated relative to the T0 baseline to illustrate the overall trajectory from 6-months post-injury through the entire intervention period.

### 2.3 Inclusion and exclusion criteria

To ensure appropriate participant selection and applicability of results to a specific population of spinal cord injury patients, rigorous inclusion and exclusion criteria were established. The patient needed to be between 18 and 50 years old - an age range selected to ensure physical maturity for exoskeleton use while minimizing age-related variables. Diagnosis of traumatic spinal cord injury between T12-L3 levels was determined through clinical and imaging criteria, with AIS C or D classification indicating incomplete injury with partial function preservation below the neurological level. Clinical and cardiovascular stability were essential requirements to ensure safety during training sessions, assessed through complete physical examinations and, when necessary, complementary cardiological evaluation. Cognitive capacity was verified using the Mini-Mental State Examination (MMSE; score=27/30) to ensure protocol comprehension and safety during exoskeleton use.

Exclusion criteria considered factors that could compromise patient safety or interfere with result interpretation. Patients with severe osteoporosis (defined by T-score below −3.0 on previous bone densitometry) were excluded due to increased fracture risk during exoskeleton training. Fixed joint contractures in lower limbs preventing proper device positioning also represented exclusion criteria, as did recent pathological fractures (within six months) that could limit weight-bearing capacity during rehabilitation. Decompensated cardiopulmonary comorbidities (NYHA Class III/IV heart failure or severe chronic obstructive pulmonary disease) were considered contraindications due to the exertion required during sessions. Finally, use of medications significantly interfering with bone metabolism (e.g., corticosteroids >5 mg/day for >3 months) also constituted exclusion criteria to avoid confounding densitometry data interpretation.

The study patient fully met all inclusion criteria without presenting any exclusion factors. His clinical condition was stable at inclusion, with no evidence of active infections or acute spinal injury complications. The documented initial anthropometric profile included weight 62.4 kg, height 1.71 m, and BMI 21.3 kg/m^2^ - within parameters considered appropriate for safe exoskeleton use. Current medication was limited to macrodantin 100 mg/day for urinary infection prophylaxis, with no drugs interfering with study parameters. His surgical history included only an exploratory laminectomy performed in January 2023, with no other relevant interventions.

### 2.4 Detailed neurological assessment

The initial comprehensive neurological evaluation documented grade 4 muscle strength on the Medical Research Council (MRC) scale for muscles innervated by L2-L3, characteristic of incomplete injury with partial motor function preservation. Sensory function remained intact up to L3, with hypesthesia below this level - a pattern consistent with both the trauma mechanism and imaging findings. Bladder and bowel functions exhibited neurogenic patterns but were adequately managed through intermittent catheterization and a specific bowel protocol. Regarding mobility, the patient was wheelchair-dependent but retained residual capacity for short-distance ambulation using a walker and orthoses, a significant factor that positively influenced his adaptation to the exoskeleton.

This detailed clinical profile, documented through complete multidisciplinary assessment, proved fundamental for developing the individualized rehabilitation intervention. The rigorous participant selection process, following clearly defined parameters, ensured not only patient safety during the study but also the internal validity of obtained results, allowing conclusions to be extrapolated with greater reliability to similar populations of incomplete paraplegia patients undergoing robotic exoskeleton rehabilitation programs. The study's methodologically rigorous approach strengthens its potential contribution to scientific literature in neurological rehabilitation, providing robust data to guide clinical practice and future research in this emerging field.

### 2.5 Body composition and bone density assessment

Body composition and bone mineral density were evaluated through dual-energy x-ray absorptiometry (DXA) using the GE Lunar iDXA system, recognized as the gold standard for such assessments according to current International Society for Clinical Densitometry (ISCD) guidelines (7). All scans were performed by an ISCD-certified technician following standardized protocols established by this scientific society. To ensure measurement accuracy, the equipment underwent daily calibration per manufacturer specifications, supplemented by weekly quality controls using anthropomorphic phantoms.

Three primary regions of interest were evaluated: the lumbar spine (L1-L4 segments), total femur (proximal region), and whole-body composition, following the assessment protocol recommended for spinal cord injury patients. This multidimensional approach enabled an integrated analysis of bone and tissue mass distribution, yielding the following quantitative parameters.

Bone mineral density (BMD) was expressed in grams per square centimeter (g/cm^2^), representing bone mineral content per unit area, as per methodology validated in recent studies. T-scores and Z-scores were calculated by comparing patient values with reference population databases, adjusted for age, sex, and ethnicity, following updated diagnostic criteria from the World Health Organization and ISCD.

Body composition analysis was performed segmentally, with absolute (kilograms) and percentage quantification of lean mass and fat mass using specialized algorithms for neuromuscular impairment populations. Particular attention was given to visceral fat quantification (expressed in grams), a metabolically significant parameter in spinal cord injury, assessed through the equipment's CoreScan™ software which employs mathematical models validated for SCI populations.

All results were interpreted by a board-certified bone densitometry specialist, accounting for the unique metabolic and functional characteristics of spinal cord injury patients.

### 2.6 Muscle and functional assessment

Muscle function evaluation was performed using isokinetic dynamometry (CYBEX Humac Norm system) following protocols established by the Brazilian Society of Sports and Exercise Medicine (8). Tests were conducted by a blinded evaluator specialized in neurological rehabilitation with international certification in isokinetic assessment. The equipment was calibrated daily according to manufacturer specifications to ensure measurement accuracy. The assessment focused on primary muscle groups involved in gait: hip extensors and flexors, knee extensors and flexors, and ankle dorsiflexors and plantar flexors. The protocol consisted of 5 maximal repetitions at 60°/s for each muscle group, with 2-minute rest intervals between sets to prevent fatigue. The following parameters were analyzed: peak torque (Nm), total work (J), average power (W), and bilateral deficit (%) (8).

This comprehensive evaluation protocol allowed for precise quantification of muscle performance characteristics relevant to gait rehabilitation. The isokinetic dynamometry provided objective, reproducible measurements of strength and power parameters essential for tracking rehabilitation progress. The standardized testing conditions, including strict adherence to angular velocity (60°/s) and rest intervals, ensured reliable comparisons across assessment time points. The inclusion of bilateral deficit measurement was particularly valuable for identifying and monitoring asymmetries in muscle function between limbs, a critical factor in gait retraining for spinal cord injury patients (8).

The blinded assessment approach minimized potential observer bias, while the evaluator's specialized training in both neurological rehabilitation and isokinetic testing guaranteed proper protocol execution. Daily equipment calibration maintained measurement precision throughout the study period, a crucial factor given the sensitivity required to detect subtle neuromuscular changes in this population. The selected muscle groups represent the key kinetic chain components for upright mobility, making their evaluation particularly relevant for exoskeleton-assisted gait rehabilitation outcomes.

The standardized parameters (peak torque, total work, and average power) provide complementary information about different aspects of muscle performance: peak torque reflects maximal strength capacity, total work indicates endurance characteristics, and average power may suggest the ability to generate force rapidly - all essential components for functional mobility. The bilateral deficit calculation enabled quantification of interlimb coordination, an important consideration for developing targeted rehabilitation strategies to improve gait symmetry. Bilateral deficit was calculated as: [(Right Peak Torque−Left Peak Torque)/Right Peak Torque] × 100 (8).

### 2.7 Functional assessment and rehabilitation program

The functional assessment protocol included administration of the AIS Impairment Scale by a board-certified physiatrist using the revised 2019 international standards (1), along with the spinal cord injury-adapted Functional Independence Measure (FIM) (9, 10). Gait evaluation consisted of the Timed Up and Go (TUG) test (10) following validated protocols for paraplegia. Patient satisfaction was measured using the Quebec User Evaluation of Satisfaction with Assistive Technology (11).

The rehabilitation program combined robotic exoskeleton training with conventional physiotherapy over six months. The exoskeleton component consisted of 20 supervised sessions using the Atalante exoskeleton (Wandercraft), conducted twice weekly. Each 60-minute session included 20 min for setup, 20 min of walking, 10 min of squats, and 10 min of recovery. Throughout these sessions, robotic assistance levels were progressively reduced from 100% assistance to −20% resistance. Concurrent conventional therapy, delivered on non-exoskeleton days, addressed residual strength deficits through resisted knee extensions (3 sets of 10 reps at 60% 1-RM) and functional mobility via overground gait training with KAFOs (twice weekly, 10 m walks). During the functional assessment walks at T1 (pre-intervention), T2 (mid-intervention), and T3 (post-intervention), the exoskeleton assistance levels were set at approximately 100%, 50%, and −20% resistance, respectively. Assistance was applied symmetrically to both limbs, with no programmed left-right differences.

Comprehensive safety monitoring was implemented, including continuous assessment of vital signs (oxygen saturation, heart rate, and blood pressure), Borg scale ratings, documentation of adverse events, skin integrity checks, and patient satisfaction measures.

### 2.8 Data analysis methodology

Given the single-case design, data are presented descriptively to illustrate longitudinal trajectories. Changes are expressed as raw differences, percentage change from baseline (Δ%), and within-subject effect sizes (Cohen's d). Associations between variables are described using correlation coefficients (r) as measures of effect magnitude, without inferential testing. All analyses are framed as exploratory observations of within-subject change.

## 3 Results

The study outcomes are presented in Tables 1–3 below, demonstrating multidimensional changes across functional, musculoskeletal, and metabolic parameters following robotic exoskeleton rehabilitation.

**Table 1:** Longitudinal isokinetic dynamometry outcomes during robotic exoskeleton rehabilitation.

Parameter	T0 (Baseline)	T1 (Pre)	T2 (Mid)	T3 (Post)	Δ% (T3 vs. T0)	Within-subject effect size (d)
Peak torque (Nm)						
R hip extension	104 ± 5.2	57 ± 3.1 (−45.2%)	69 ± 4.8 (+21.1%)	84 ± 6.0 (+21.7%)	−19.2%	1.12
L hip extension	92 ± 4.8	62 ± 3.5 (−32.6%)	46 ± 3.2 (−25.8%)	49 ± 3.4 (+6.5%)	−46.7%	2.03
Bilateral deficit	12% ± 1.8	9% ± 1.2	33% ± 2.9	42% ± 3.5	+250%	3.41
Total work (J)						
R hip extension	437 ± 22	320 ± 18 (−26.8%)	266 ± 15 (−16.9%)	301 ± 17 (+13.2%)	−31.1%	0.97
L hip extension	281 ± 14	267 ± 13 (−5.0%)	207 ± 12 (−22.5%)	201 ± 11 (−2.9%)	−28.5%	1.15
Average power (W)						
R hip extension	82 ± 4.1	81 ± 4.0 (−1.2%)	63 ± 3.5 (−22.2%)	63 ± 3.5 (0%)	−23.2%	0.89

Longitudinal isokinetic dynamometry outcomes (CYBEX Humac Norm system) during robotic exoskeleton rehabilitation in incomplete L2 paraplegia (AIS C). Data shown as mean ± SD of 5 maximal repetitions. Δ% indicates percentage change from T0 (Baseline) to T3 (Post-Intervention). Within-subject effect sizes (Cohen's d) are calculated for the change from T0 (Baseline) to T3 (Post-Intervention). Bilateral deficit calculated as [(R-L)/R] × 100.

**Table 2:** Longitudinal body composition and bone mineral density changes during robotic exoskeleton rehabilitation.

Parameter	T0 (Baseline)	T3 (Post)	Absolute Δ	Δ% (T3 vs. T0)	Clinical thresholds
Body composition					
Weight (kg)	62.4	68.2	+5.8	+9.3%	-
Lean mass (kg)	39.78 ± 0.8	41.41 ± 0.9	+1.63	+4.1%	-
Lean mass (% body weight)	66.22%	61.5%	−4.72%	−7.1%	Normal range: >65% (young men)
Fat mass (kg)	17.23 ± 0.5	23.39 ± 0.6	+6.16	+35.7%	-
Fat mass (% body weight)	30.2%	36.1%	+5.9%	+19.5%	Obesity: >25% (men)
Visceral fat (g)	91 ± 12	418 ± 25	+327	+359%	High risk: >300g
Android/gynoid ratio	0.74	0.89	+0.15	+20.3%	Cardiometabolic risk: >0.85
Bone mineral density					
Lumbar spine (L1-L4) (g/cm^2^)	0.88 ± 0.03	1.32 ± 0.04	+0.44	+50%	Osteoporosis: <0.8 g/cm^2^
Lumbar T-score	−2.6	0.8	+3.4	-	Normal: >−1.0
Femur total (g/cm^2^)	0.679 ± 0.02	0.917 ± 0.03	+0.238	+35.1%	-
Femur T-score	−3.3	−1.3	+2.0	-	Severe osteoporosis: <−2.5

Data presented as mean ± standard deviation where applicable. Δ = change from baseline. Reference thresholds from ISCD guidelines (2023).

**Table 3:** Functional outcomes before and after exoskeleton intervention.

Metric	T0 (Baseline)	T3 (Post)	Δ% (T3 vs. T0)	Within-subject effect size (d)	MCID threshold
Neurological					
AIS motor (L2-L3)	4/5	5/5	+25%	1.2	+1 grade
Light touch score (/112)	78	84	+7.7%	0.8	+5 points
Mobility					
10MWT (m/sec)*	0.28 ± 0.05	0.41 ± 0.06	+46.4%	1.7	+0.05 m/sec
TUG (sec)	47.7 ± 2.5	30.0 ± 2.0	−37.1%	1.4	−3.6 s
WISCI II level	8	12	+4	-	+1 level
FIM (score)	68	96	+41.2%	2.4	+22 points
Endurance					
6MWT (meters)	182 ± 15	300 ± 20	+64.8%	2.1	+45.8m

10MWT, 10-meter walk test (9); TUG, timed up and go (10); WISCI II level, walking index for spinal cord injury (11); FIM, functional independence measure; MCID, minimal clinically important difference (SCI-specific thresholds from 12). Data: mean ± SD unless noted. Effect sizes: Cohen's d (small≥0.2, medium≥0.5, large≥0.8).

A visual synthesis of the key longitudinal trends reveals distinct patterns. The recovery of right hip extensor torque followed a biphasic trajectory: a sharp decline from T0 to T1 (−45.2%), followed by progressive recovery through T2 and T3, culminating in a value at T3 that remained 19.2% below the original baseline. In contrast, left hip extensor torque showed a near-monotonic decline across all timepoints. Consequently, the bilateral deficit, a measure of interlimb asymmetry, increased steadily from 12% at T0 to 42% at T3. Metabolic and structural changes presented a stark contrast: lumbar bone mineral density exhibited a substantial linear increase (+50% by T3), while visceral adipose tissue accumulated exponentially over the same period, culminating in a 359% increase. Functional mobility metrics mirrored the positive torque trend, with Timed Up and Go time decreasing and 6-minute walk distance increasing progressively across T0 to T3. These co-occurring yet divergent trajectories underscore the multidimensional nature of the response.

The intervention exhibited an excellent safety profile, with only two transient adverse events (mild cutaneous erythema resolving within 24 h without intervention). Adherence to the protocol was high (92 ± 3% of sessions completed), as recorded in therapy logs. No serious adverse events (e.g., fractures, falls) or dropouts occurred during the study period.

The patient showed asymmetric responses, with right-sided improvement but progressive left-sided decline. While functional mobility measures improved, these occurred alongside concerning metabolic changes and worsening bilateral asymmetry.

Isokinetic dynamometry revealed a two-phase response: initial 45.2% decline in right hip extensor torque (104→57 Nm) post-injury, followed by progressive recovery during exoskeleton training, with 21.7% improvement in the final phase (69→84 Nm). Despite this, torque remained 19.2% below baseline (d = 1.12), suggesting incomplete neuromuscular adaptation. Bilateral deficit worsened from 12% to 42% (d = 3.41), showing an asymmetric recovery. This demonstrates the biphasic pattern of recovery: acute post-injury decline (Torque D: −45.2%) followed by progressive improvement with exoskeleton (+21.7%), but without return to baseline (−19.2%).

A strong inverse association was observed between right hip extensor torque and TUG performance (r = −0.71), indicating greater strength predicted faster mobilityAn exploratory regression analysis suggested an association between baseline muscle strength and final functional independence (FIM score; unstandardized *β* = 0.42), accounting for 28.5% of outcome variance in the multivariate model (*R^2^* = 0.68). Functional independence, as measured by the FIM, improved by 41.2% (from 68 to 96 points).

## 4 Discussion

The patient's trajectory demonstrates that robotic exoskeleton rehabilitation was associated with a mix of beneficial and adverse multisystem changes in patients with incomplete paraplegia, confirming previous findings shows this technology's potential for neuroplastic and functional modulation (7). The 50% increase in lumbar bone mineral density (from 0.88 to 1.32 g/cm^2^) exceeds results reported with conventional therapies such as functional electrical stimulation (FES) or whole-body vibration, which typically achieve gains of 8%–20% over similar periods (13).

These findings suggest that axial loading through exoskeleton training may more efficiently activate bone mechanotransduction pathways, potentially via osteocyte stimulation and Wnt/*β*-catenin signaling activation, as supported by mechanobiology studies in unloading models (14). The observed bone density changes likely reflect the unique biomechanical loading patterns generated during exoskeleton-assisted gait, which appear superior to non-weight-bearing modalities for triggering osteogenic responses in neurologically impaired patients.

Although, the worsening of bilateral torque deficit (from 12% to 42%) reveals a critical limitation: current exoskeletons may amplify pre-existing muscle asymmetries in incomplete spinal cord injuries. This phenomenon may be related to asymmetric activation of residual neural circuits below the injury level. Furthermore, the 70.8% reduction in left knee flexor power (from 24W to 7W) suggests that robotic training alone may be insufficient to prevent atrophy in partially innervated muscles. This can show the need for protocol adjustments to ensure more balanced muscle activation (15, 16). The maintained power deficit (−23.2%) despite torque improvement may explain the patient's reported fatigue during prolonged walking, indicating need for endurance-focused training phases.

Functional improvements were notable, including a four-level progression on the WISCI II scale (from 8 to 12) – exceeding typical gains with conventional rehabilitation. Furthermore, the strong inverse relationship between right hip extensor torque and TUG performance (r = −0.71) supports the idea that preserved lumbar neural circuits (L2-L3) are a key substrate for functional recovery with robotic training. This relationship also suggests hip extensor torque may serve as a useful biomarker for monitoring rehabilitation efficacy in similar patients (6). This progression is a new finding, where patients with baseline muscle strength ≥4 on the AIS scale demonstrated greater responsiveness to exoskeleton training.

Our results demonstrate a strong inverse relationship between hip torque generation and TUG performance time (r = −0.71), supporting emerging evidence that preservation of lumbar neural circuits (particularly at L2-L3 levels) serves as a key predictor of locomotor recovery potential. This finding aligns with Human neurophysiological data confirming the importance of hip flexor torque as a biomarker for functional recovery after SCI (17).

The strong negative correlation between hip torque and TUG time (r = −0.71) supports the hypothesis that preserved neural circuits at L2-L3 play a crucial role in robotic rehabilitation success. The 41.2% improvement in Functional Independence Measure scores (from 68 to 96 points) carries important clinical and socioeconomic implications, as each 10-point FIM gain reduces annual caregiver costs, potentially justifying the exoskeleton's substantial initial investment (18).

DXA revealed a clinically concerning 359% increase in visceral fat (91g→418 g), exceeding metabolic risk thresholds (>300 g) despite 4.1% lean mass gain. absolute lean mass increased 4.1% (39.8→41.4 kg), Its relative contribution to body weight decreased from 66.2% to 61.5% due to disproportionate fat accumulation (30.2%→36.1% body fat). This paradoxical increase in visceral adipose tissue was observed during the intervention period alongside improved BMD (+50%), suggesting dissociation between osteogenic and metabolic responses to exoskeleton training. This study's findings, interpreted in the context of existing literature, provide new insights into the mechanisms of robotic exoskeletons in spinal cord injury rehabilitation, particularly regarding autonomic modulation and metabolic challenges. The substantial increase in visceral fat coincident with functional improvement suggests a possible dissociation between motor benefits and metabolic adaptations in paraplegic patients. While not measured in this case, this phenomenon may be linked to persistent autonomic dysfunction post-injury, which disrupts adipose tissue regulation and insulin sensitivity (5). Repetitive exoskeleton stimulation can modulate residual sympathetic activity, but it remains insufficient for normalizing energy metabolism in patients with thoracic or lumbar injuries. These results highlight the critical need to integrate metabolic management strategies with robotic rehabilitation to achieve comprehensive patient outcomes. Importantly, unmeasured factors such as dietary habits and free-living physical activity may have contributed substantially to this metabolic change.

The 50% increase in lumbar BMD, while clinically significant, doesn't fully resolve bone fragility concerns. Longitudinal studies must determine if this improvement reduces fracture risk. This paradox demands cautious load progression and suggests combining robotic training with osteoanabolic therapies like teriparatide, which demonstrated synergistic effects with exercise in recent post-SCI osteoporosis trials (18). The improved BMD, though promising, requires careful clinical interpretation given the complex relationship between bone density, microarchitecture, and fracture risk in neurogenic osteoporosis. Current evidence indicates the need for: 1) Close monitoring during early rehabilitation phases, 2) Adjunctive bone-forming medications, and 3) Personalized loading protocols that balance osteogenic stimulation with structural safety. These findings highlight both the potential and limitations of robotic rehabilitation for addressing post-SCI bone loss, emphasizing the importance of multimodal approaches to fracture prevention.

Additionally, the integration of epidural stimulation with exoskeletons is being tested in pioneering studies, with preliminary results indicating improvements in muscle activation symmetry and postural control (16). These advances may help overcome the limitations observed in our study, particularly in patients with asymmetric patterns of neurological preservation.

From a translational perspective, the data from this study support the feasibility of exoskeletons as a rehabilitation tool but highlight the importance of a multidisciplinary approach. The inclusion of nutritional, pharmacological, and neuromodulation strategies may enhance the benefits of robotic training, especially in patients with incomplete injuries. The development of predictive biomarkers, such as serum levels of irisin and neurofilaments, could help identify individuals with the greatest potential for treatment response. Additionally, cost-effectiveness economic analysis becomes crucial, given the high initial investment required for exoskeletons. Prospective studies should assess whether the observed functional gains translate into sustained reductions in healthcare costs and long-term improvements in quality of life (3, 4).

Several limitations must be acknowledged in this case report. First, the single-subject design inherently limits generalizability of findings to broader SCI populations. Second, the combined intervention approach (exoskeleton plus conventional therapy) precludes isolation of the exoskeleton's specific effects. Third, the 6-month intervention period with only 1-month follow-up provides limited insight into long-term outcomes. Fourth, metabolic assessments were restricted to DXA-derived measures without endocrine or inflammatory marker evaluation. Fifth, autonomic function was not systematically assessed despite its potential relevance to observed metabolic changes. Finally, the lack of a control period or multiple baseline assessments weakens causal inferences about the intervention's effects. These limitations highlight the need for larger controlled studies with extended monitoring periods. It is important to note that the discussions of metabolomic changes, autonomic modulation, and myokine activity are speculative interpretations drawn from the broader scientific literature. These specific biomarkers were not measured in the present case report, and thus the proposed mechanisms remain hypotheses for future investigation.

As recommended by CARE guidelines, incorporating the patient's own experiences would have enriched this case report. While we documented quantitative outcomes and clinician observations, systematic collection of patient-reported outcomes (e.g., quality of life measures, satisfaction ratings, or personal recovery narratives) was omitted. The patient did report subjective improvements in “standing endurance” and “confidence during transfers” during clinical interactions, but these were not captured through standardized instruments like the SCI-QOL or qualitative interviews. Future case studies should prioritize mixed-methods approaches that combine objective measures with structured patient narratives to provide more holistic understanding of rehabilitation experiences. This limitation particularly affects interpretation of the visceral fat accumulation, as dietary habits and personal activity patterns outside sessions were not recorded.

This analysis underscores the complexity of exoskeleton-based rehabilitation, which must evolve beyond a purely motor-focused approach to incorporate integrated strategies addressing post-spinal cord injury metabolic, autonomic, and musculoskeletal dysfunctions. These observations may inform the future research focused on personalized protocols and combined interventions.

## 5 Conclusions

This case report documents the multidimensional response of an 18-year-old male with chronic L2 AIS C paraplegia to a 6-month combined rehabilitation program. The patient demonstrated clinically meaningful functional improvements, including enhanced gait capacity (300 m with crutches) and faster transfer times (37% reduction in TUG), which were maintained at short-term follow-up. Notably, these functional gains occurred alongside paradoxical physiological changes - while lumbar bone density showed substantial improvement (50% increase), the intervention coincided with concerning metabolic alterations including a 359% rise in visceral adipose tissue. The development of significant neuromuscular asymmetry (worsening bilateral deficit from 12% to 42%) despite overall functional progress underscores the complexity of rehabilitation in incomplete spinal cord injuries. Several limitations temper interpretation, including the inability to isolate effects of specific intervention components, absence of long-term outcome data, and lack of structured patient-reported experience measures as recommended by CARE guidelines. For clinical practice, this case highlights the importance of monitoring both functional and metabolic parameters during rehabilitation, and suggests patients with similar profiles may benefit from targeted asymmetry correction and nutritional interventions. As a single-subject observation, these findings cannot support generalized conclusions about treatment efficacy, but they provide valuable hypothesis-generating data regarding the dissociation between functional recovery and metabolic health in incomplete paraplegia rehabilitation. Future research should prioritize integrated assessment protocols that capture both physical and metabolic outcomes, along with standardized patient experience measures.

## Data Availability

The datasets presented in this study can be found in online repositories. The names of the repository/repositories and accession number(s) can be found in the article/Supplementary Material.
